# Our Contemporaries

**Published:** 1913-08

**Authors:** 


### OUR CONTEMPORARIES

---

The *Smart Set,* which is not so much immoral as cynical, in its May issue questions the extent of white slavery on account of the abundant supply of free labor in this nefarious industry.

---

The *Texas Medical Journal, April,* while maintaining a special department of Eugenics and Sex Hygiene, under the editorship of Mrs. F. E. Daniel, wife of the editor, contains a refreshingly sane editorial on the tendency to overdo sex education in the young. There is mentioned the seven-year-old son of a university professor who entertained callers by describing the lacerations which his mother suffered during child-birth.

---

THE DOCTOR'S FACTOTUM, June, is an interesting quarterly published by the N. Y. Pharmacal Assn., Arlington Chemical Co. and Palisades Mfg. Co. of Yonkers. It contains a selection of articles from medical literature and a collection of humorous items and poems bearing on medicine.

METHODS OF COMPULSORY MEDICINE ADVOCATES.—Dr. Wiley, in an address delivered at Dayton, Ohio, October 29, according to the Dayton newspapers, used these words:

"In Indiana, Dr. Perry, with an appropriation of $100,000, has done wonderful work. He has stamped out at least one infectious disease, namely, diphtheria. The State Board of Health keeps the County Board of Health supplied at all times with serum, and as soon as a case of diphtheria appears it is immediately stamped out. *There has not been a death from diphtheria in Indiana in the last four years.*"

According to the Indiana State Board of Health Bulletins there were, in 1909, 338 deaths from diphtheria; in 1910, 381 deaths; in 1911, 343 deaths, and during the first eight months of 1912, 151 deaths, or a total of 1,213 deaths from this disease in something less than four years.

These methods of impressing the public ear with the benefits of antitoxin are not at all uncommon, and to listen to such addresses as Dr. Wiley's one would suppose that antitoxin had succeeded in entirely eliminating diphtheria, when, as a matter of fact, it has done nothing of the kind. In 1904 and 1905 the deaths from diphtheria in Indiana were respectively 314 and 366. If antitoxin or the serum "supplied at all times" by the Indiana Board of Health is doing such wonders, why this increase in the mortality during the past four years?

The above is from the *Medical Century* of February, 1913, which is hostile to what has been termed the Medical Trust by its enemies. We do not believe that any cause is so worthy as to justify a mis-statement of facts, but it occurs to ask whether it is a fact that Dr. Wiley made the statement attributed to him.

---

**Little Conveniences and Economies.**

To protect the flame of the Bunsen burner from drafts and thus to secure uniform temperature in incubating, evaporating, extracting fats, etc., inclose the burner with a mica chimney. One way to do this is to fasten the base of the burner to a fairly large block of wood with three or four screw-eyes. Set over the stem of the Bunsen a tall chimney support such as used with a mantel gas light. The projections from this support can be fastened by a fine wire or picture cord passing through the screw-eyes.

Faeces, stomach contents, etc., pass through ordinary filters very slowly. Time may be saved by preliminary filtration through white paper napkin. Test the napkin with the various indicators to be used, to make sure that there will be no interference with subsequent tests. Saving of time in filtration means increased accuracy, on account of the fermentative and putrefactive changes liable to occur with delay.

For titration, a white porcelain receptacle affords a better background for colors than glass, even when held against a white surface. A rather narrow, fairly deep receptacle is more convenient than a shallow dish. An elcaya cream jar may be used.

An old lined glove is a satisfactory test-tube holder. The ulnar portion may be removed for the sake of coolness.

A water power centrifuge should be securely mounted and requires a good head of water. One way is to place a short piece of *plank* across the faucet end of a bath tub or diagonally from this end to the wall side, fastening the plank to the tub by metal screw clamps. To render the plank more secure, chisel notches for the clamps. Before screwing the centrifuge to the plank, make sure that it is located so that the revolving tubes will strike nothing and will not even be in the way.

